# Plausible Minimal Substrate for Erm Protein

**DOI:** 10.1128/AAC.00023-20

**Published:** 2020-08-20

**Authors:** Hak Jin Lee, Young In Park, Hyung Jong Jin

**Affiliations:** a Department of Life Science, Korea University Graduate School, Seoul, Republic of Korea; b Department of Bioscience and Biotechnology, The University of Suwon, Whasung City, Republic of Korea

**Keywords:** Erm protein, antibiotic resistance, methylation, minimal substrate

## Abstract

Erm proteins methylate a specific adenine residue (A2058, Escherichia coli coordinates) conferring macrolide-lincosamide-streptogramin B (MLS_B_) antibiotic resistance on a variety of microorganisms, ranging from antibiotic producers to pathogens. To identify the minimal motif required to be recognized and methylated by the Erm protein, various RNA substrates from 23S rRNA were constructed, and the substrate activity of these constructs was studied using three Erm proteins, namely, ErmB from *Firmicutes* and ErmE and ErmS from *Actinobacteria*. The shortest motif of 15 nucleotides (nt) could be recognized and methylated by ErmS, consisting of A2051 to the methylatable adenine (A2058) and its base-pairing counterpart strand, presumably assuming a quite similar structure to that in 23S rRNA, an unpaired target adenine immediately followed by an irregular double-stranded RNA region. This observation confirms the ultimate end of each side in helix 73 for methylation, determined by the approaches described above, and could reveal the mechanism behind the binding, recognition, induced fit, methylation, and conformational change for product release in the minimal context of substrate, presumably with the help of structural determination of the protein-RNA complex. In the course of determining the minimal portion of substrate from domain V, protein-specific features could be observed among the Erm proteins in terms of the methylation of RNA substrate and cooperativity and/or allostery between the region in helix 73 furthest away from the target adenine and the large portion of domain V above the methylatable adenine.

## Abstract (precis)

Erm proteins methylate a specific adenine residue (A2058, Escherichia coli coordinates) conferring macrolide-lincosamide-streptogramin B (MLS_B_) antibiotic resistance on a variety of microorganisms, ranging from antibiotic producers to pathogens. To identify the minimal motif required to be recognized and methylated by the Erm protein, various RNA substrates from 23S rRNA were constructed, and the substrate activity of these constructs was studied using three Erm proteins, namely, ErmB from *Firmicutes* and ErmE and ErmS from *Actinobacteria*.

## INTRODUCTION

Erm proteins mono- or dimethylate a specific adenine residue (A2058, Escherichia coli coordinates) on an exocyclic amino group to reduce the affinity of macrolide-lincosamide-streptogramin B (MLS_B_) antibiotics, thereby conferring resistance on the microorganisms harboring Erms (1, 2). While dimethylation induces high resistance to all MLS_B_ antibiotics, monomethylation is effective against the action of lincosamide but confers only slight protection against the antimicrobial activity of macrolide (3–5). Domain V of 23S rRNA has been considered to contain all the structural elements required to be recognized and methylated by Erm proteins (6–8). Truncation of a variety of helices and loops of domain V led to the identification of smaller RNA structures methylated by Erm proteins, ranging from as small as 27-nucleotide (nt) RNA (9), 24-nt RNA (10), and 32-nt RNA (11) to 41-nt RNA (12). Almost 40 different *erm* methyltransferase genes have been isolated and characterized from diverse sources, ranging from pathogens to antibiotic-producing actinomycetes (1, 2, 13, 14). Despite the diversity of sources, the significant homology among Erm proteins and their common functional role in methylating the specific adenine residue at position N^6^ suggest that they could assume similar structures (11, 15) and originate from a common ancestor (2, 16). Furthermore, the methylatable adenine (A2058) is located in the peptidyl transferase circle in domain V of 23S rRNA, which has been highly conserved over the course of evolution, presumably because of its functional role as the active site of the ribosome (17, 18). From these observations, it has been inferred that the methylation at A2058 by Erm proteins is performed by a specific interaction between conserved amino acids in the protein and the conserved nucleotide motif in domain V, which could be supported by the fact that Erm proteins from Gram-positive (ErmS and ErmE) and -negative bacteria (ErmC) share a complete substrate, domain V, and that a similar structural motif of minimal substrate for these Erm proteins, consisting of helix 73 and single-stranded loop region containing the methylatable adenine, needs to be methylated. If more detailed aspects of methylation are considered, biological diversity might be reflected in this very important antibiotic resistance mechanism, as this mechanism is adopted to allow survival, a matter of “to be or not to be” in the presence of antibiotics. Each Erm protein situates itself in its own environment to meet the requirements of the cell expressing it. Therefore, there could be some diversity in methylation in accordance with why the protein is present at a particular site. In fact, some discrepancies have been observed in the function of A2051. In particular, methylation is essential in ErmE (9); but, decreased activity for ErmS (6) was found upon deletion of A2051, whereas it was methylated to a lesser extent in the presence of A2051 with ErmC (11) in the context of a similar small substrate size, even though they used different rRNA fragments, E. coli, and Bacillus subtilis and there was some sequence variation. Furthermore, the reported minimal substrates for ErmC´, ErmE, and ErmS differ from each other in terms of size and are 32-nt RNA (11), 24- or 27-nt RNA (9, 10), and 41-nt RNA (12), respectively. These observed variations could be attributable to the different pairs of the Erm protein and its cognate RNA substrate investigated, suggesting that each Erm protein could recognize somewhat different sets of nucleotides in order to recognize and methylate the rRNA substrate. As a first step to elucidate the difference in rRNA methylation among the Erm proteins and to define the primary determinant of specificity in order to facilitate the study of the enzymatic reaction in detail, with the aim of obtaining a comprehensive understanding of the protein-RNA interaction, efforts have been made to identify what minimal portion of domain V of bacterial 23S rRNA is required for methylation at A2058 by Erm proteins. ErmS, ErmE, and ErmB were focused on in this study for the following reasons. A comprehensive phylogenetic analysis generated a tree showing early bifurcation of *Firmicutes* and *Actinobacteria* (2). Therefore, representatives of each of these branches were chosen here, namely, ErmS and ErmE from *Actinobacteria* and ErmB from *Firmicutes*. Moreover, in addition to the main body of the protein, ErmS harbors the longest N-terminal end region, whereas ErmE has the longest C-terminal end region, possibly reducing the required nucleotides in substrate RNA to be minimized more.

## RESULTS

### Identification of the end nucleotide(s) in helix 73 furthest away from the methylatable adenine to be methylated by Erm protein with truncation of domain V.

**FIG 1:**
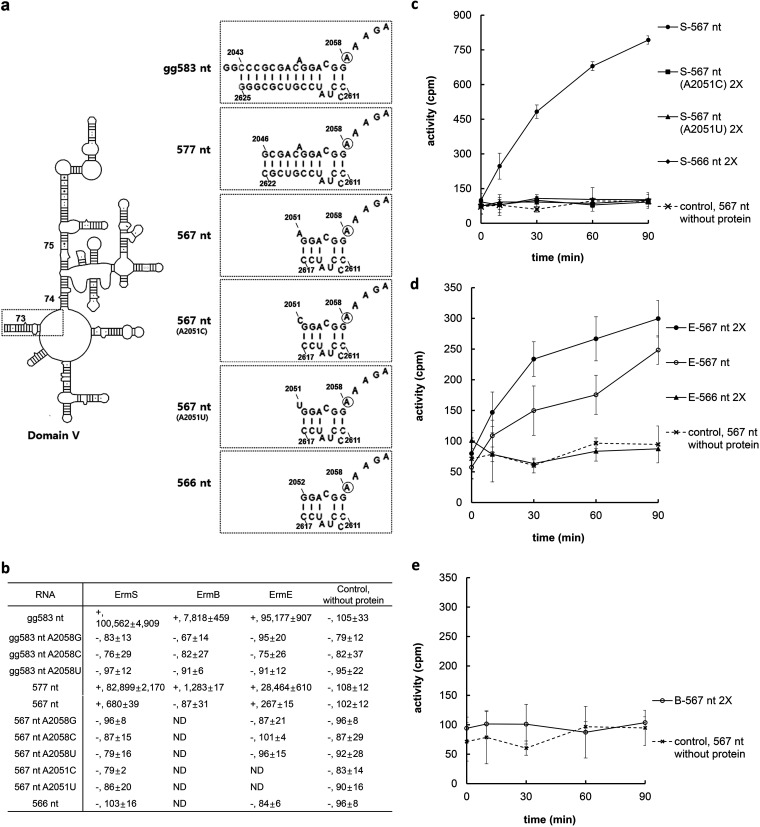
Determining the end nucleotide or base pair through the deletion of helix 73 in domain V to be methylated by Erm proteins. (a) Domain V (gg583 nt) used in this study contains only the exact sequence of helix 73, without any further extension on either side, but extra GG dinucleotides were attached at the 5′ end for successful *in vitro* transcription. Three base pairs (577 nt), five base pairs (567 nt) and A2051 (566 nt) were deleted stepwise from domain V to identify the end nucleotide or base pair to be methylated by Erm proteins. To assist in finding out whether the deletion of A2051 could induce structural alteration, the A2051U and A2051C mutants of 567 nt were constructed, and their substrate activities were measured. The methylatable adenine is circled. (b) Substrate activity of domain V-related constructs associated with the used Erm proteins. Each value represents the mean of at least three independent experiments obtained after 1 h of incubation in the presence of cofactor SAM. Refer to the Results. Mutant substrates in which mutations were introduced at A2058 could not be methylated by Erm proteins to confirm the specific methylation at A2058. Also the 567-nt A2051U and A2051C mutants could not show any substrate activity at all, suggesting that the deletion of A2051 from 567 nt might not induce any structural perturbation. + and – represent positive and negative substrate activity, respectively. (c) To ensure the negative or positive substrate activity of 567 nt, 566 nt, and 567 ntA2051U and 567 ntA2051C mutants, methyl group-accepting activity was monitored over time and in the presence of double enzyme concentrations, together with a control lacking protein because of their quite low substrate activity, if it has substrate activity. Each value represents the mean of at least three independent experiments with standard deviation. 2X denotes monitoring substrate activity with double the concentration of the enzyme.

The common feature of identified minimal substrates using three different Erm proteins, ErmC´, ErmE, and ErmS, is that they should exhibit at least some or all of helix 73 and the unpaired loop region containing the methylatable adenine (9–12). As such, we attempted to define the end nucleotide or base pair on the distal side of helix 73 from the methylatable adenine to be methylated by each Erm protein, which might reveal some of the characteristic binding modes of each Erm protein. These experiments were performed in the presence of all the other domain V component nucleotides because there is a possibility that the presence of other domain V nucleotides could compensate for the loss of contacts in helix 73 by truncation (Fig. 1a). In contrast to various domain V constructs used in previous studies (6–8, 19), which contained extra nucleotides before the 5′ end of helix 73 and/or after its 3′ end, domain V harboring only the exact helix 73 was used in this study, from which gradual truncation was performed to define the end nucleotide or base pair for accepting the methyl group from *S*-adenosyl-l-methionine (SAM) with the action of Erm proteins (Fig. 1a). With that domain V of gg583 nt in Fig. 1a, ErmS showed the highest methylating activity, which was almost 13 times higher than the activity of ErmB, transferring 36.01% of SAM (3.3 pmol) under standard assay conditions. After removal of three base pairs in helix 73 from this construct (577 nt), the extent of reduction in substrate activity differed depending on the Erm protein used. The substrate activity was most severely reduced by about 84% in ErmB, whereas with ErmS, the reduction was only 18%. In addition, for ErmE, 70% activity was lost with this truncation. Further deletion of five more base pairs up to A2051 (E. coli numbering, 567 nt) left ErmB unable to methylate the resultant rRNA fragment; the other two Erm proteins still retained the methylating activity with this substrate, although less than 1% substrate activity remained with ErmS (0.01 pmol transferred out of 3.3 pmol provided in the assay medium) and ErmE (0.002 pmol out of 3.3 pmol). Truncation of A2051 could abolish the methyl group-transferring activity of all the Erm proteins, despite the presence of all the other domain V nucleotides in the substrate (Fig. 1b). These observations, especially of the retention of methylation activity of ErmE with 567 nt RNA and the loss of methylation activity of ErmB with 567 nt RNA (Fig. 1b and e) and ErmS (Fig. 1b and c) and ErmE (Fig. 1b and d) with 566 nt RNA, were confirmed using double the amount of each protein in the reaction mixture and with time under standard assay conditions because of their very low substrate activity. From these observations, 567 nt was supposed to assume the desired conformations because, with ErmS, 567 nt consistently exhibited high substrate activity and it increased reasonably with time. In addition, ErmE could increase methyl group transfer to 567 nt over time with little variation. Moreover, with double the amount of ErmE, the substrate activity of 567 nt appeared to increase accordingly. It was inferred that the deletion of the dangling nucleotide A2051 from 567 nt to make 566 nt did not induce any structural perturbation because A2051 C and A2051 U mutants did not show any substrate activities (Fig. 1a, b, and c; see below in Discussion). Furthermore, it could be concluded that methylation by Erm proteins occurred specifically at A2058 because none of the mutant substrates (A2058G, A2058C, and A2058U) exhibited any methyl group incorporation into them (Fig. 1b).

### Confirmation of end nucleotide(s) in helix 73 using small RNA substrate.

**FIG 2:**
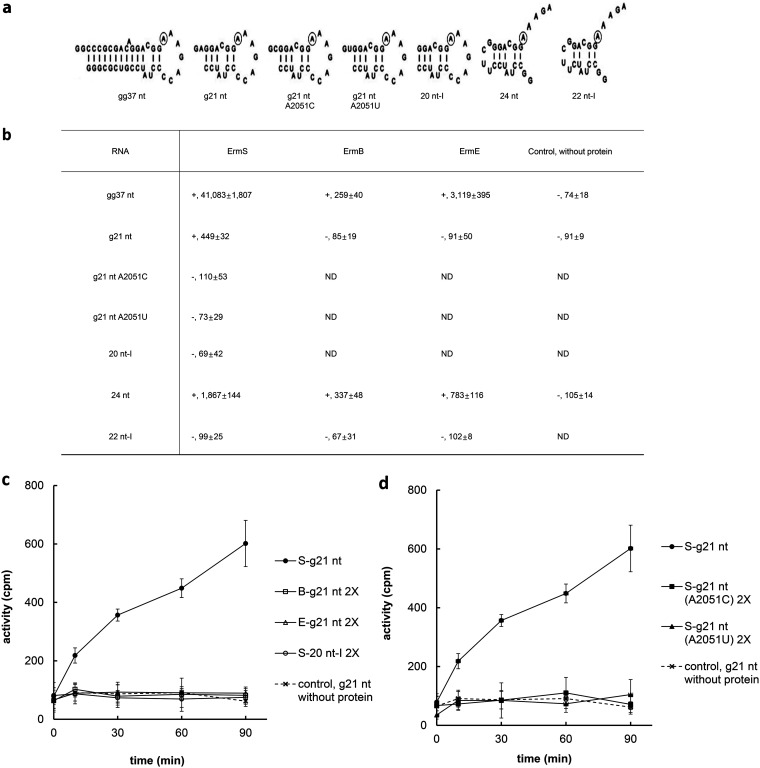
Confirming the ultimate end on the other side of the methylatable adenine in helix 73 to be methylated by Erm proteins in the context of minimal structure. (a) Loop sequence (7 nt) tethering on the methylatable adenine side contains the natural sequence **A**AAGA (bold letter denotes the methylatable adenine) connecting helix 73 and 74 and extra CC nucleotides. The loop capping the opposite side of the methylatable adenine in helix 73 was the UUCG tetraloop, which appeared to enhance the substrate activity, as shown in (b), presumably by providing stronger binding affinity to the enzymes than A2051. With the UUCG tetraloop, the other side could be unleashed. Circled is the methylatable adenine. Extra GG and G were attached to the 5′ end of gg37 nt and g21 nt and its mutants (g21 ntA2051U and ntA2051C) to produce more RNA by *in vitro* transcription. (b) The methyl group-accepting activity of tentatively minimized constructs for determining the ultimate opposite end of the methylatable adenine. The g21 ntA2051U and g21 ntA2051C mutants were employed to assist in finding out whether the deletion of A2051 might induce the structural disturbance from g21 nt. The results are the average value of three independent experiments, which were obtained after 1 h of incubation in the presence of cofactor SAM. Refer to the Results. + and – in front of each value represent positive and negative substrate activity, respectively. (c) To confirm the negative substrate activity of g21 nt to ErmB and ErmE and 20 nt-I, (d) g21 ntA2051U, and g21 ntA2051C mutants to ErmS, methyl group-accepting activity was monitored over time and in the presence of double enzyme concentrations because of their quite low substrate activity, if it has substrate activity. Each value represents the mean of at least three independent experiments with standard deviations. 2X denotes monitoring substrate activity with double the enzyme concentration. g21 nt, which is equipped with A2051 and a 7-nt loop sequence, could be methylated by ErmS, but not by ErmB and ErmE. 20 nt-I, where one base pair was deleted from g21 nt and g21 nt A2051U and A2051C mutants, could not be methylated even with ErmS.

It has been observed that there is some difference in the ability to accept methyl groups according to the length of hairpin loop tethering the methylatable adenine side of helix 73 when using ErmE (9) and ErmC´ (11). However, some length of helix 73 fragments capped with a hairpin loop (7 nt **A**AAGAcc, uppercase letters represent the natural sequence connecting stem 73 and 74, with bold **A** being the methylatable adenine, and lowercase letters being artificially added nucleotides) (Fig. 2a) has been shown to be methylated by ErmE (9, 10) and ErmC´ (11). Therefore, constructing several lengths of helix 73 capped on the methylatable adenine side with the sequence (7-nt hairpin loop) described above, their substrate activity was monitored (Fig. 2a). When the RNA fragment was equipped with the whole helix 73 sequence and tethered with a 7-nt hairpin loop on the target adenine side, all the Erm proteins employed exhibited methylating activity on this substrate (gg37 nt) (Fig. 2b). As observed above with domain V, a much smaller RNA fragment (g21 nt) truncated up to position A2051 could be methylated by ErmS, which was again consistent with the result obtained using domain V. However, with this substrate, no methyl group-accepting activity could be obtained with ErmB, consistent with the observation with domain V. For ErmE, inconsistency arose between using domain V and g21 nt as a substrate; domain V truncated up to A2051 (567 nt) showed substrate activity, but g21 nt did not. Interestingly, once A2051 was deleted, all of the Erm proteins (ErmS and ErmE) that exhibited the methyl group-transferring activity in the presence of A2051 could not methylate the RNA, irrespective of whether the truncated RNA substrate originated from domain V (566 nt) or smaller RNA substrates (20 nt-I). Deletion of A2051 from g21 nt was not supposed to induce any structural perturbation because two mutations, A2051C and A2051U, could not exhibit any substrate activity, and so, A2051 is attached dangling (Fig. 2a, b, and d; and see below in Discussion). However, when this small substrate was tethered with a UUCG tetraloop after deleting A2051 (24 nt) (Fig. 2a), the methyl group-accepting activity was greatly enhanced with all the Erm proteins tested, which was even higher than that for gg37 nt in the case of ErmB (Fig. 2b). Despite this unexpected increase in substrate activity by capping with the UUCG tetraloop, further truncation of one more base pairs toward the target adenine (22 nt-I) (Fig. 2b) appeared to completely abolish the methyl group-accepting activity. Uncertainty caused by quite low activity regarding whether there was positivity for methylation or not was overcome by the addition of double amounts of enzymes in the reaction mixture (Fig. 2c and d). In addition, specific methylation at A2058 was confirmed as well for small RNA substrates using A2058G, A2058C, and A2058U mutant substrates (Table 1).

**TABLE 1:** Demonstration of specific methylation of minimized substrates at A2058 by Erm proteins using mutant substrates

RNA substrate	Methylation result by treatment^*a*^			
	ErmS	ErmB	ErmE	Control without protein
24 nt	+	+	+	−
24 nt A2058G	−	−	−	−
24 nt A2058C	−	−	−	−
24 nt A2058U	−	−	−	−
g21 nt	+	−	−	−
g21 nt A2058G	−	ND^*b*^	ND	ND
g21 nt A2058C	−	ND	ND	ND
g21 nt A2058U	−	ND	ND	ND
20 nt-II	+	ND	ND	−
20 nt-II A2058G	−	ND	ND	ND
20 nt-II A2058C	−	ND	ND	ND
20 nt-II A2058U	−	ND	ND	ND
Annealed 15 nt	+ (18°C)	ND	ND	−
Annealed 15 nt A2058C	− (18°C)	ND	ND	ND
Annealed 15 nt A2058U	− (18°C)	ND	ND	ND

a Positive methylation at A2058 is denoted as + and – represents no methylation. Corresponding positive values can be found in Fig. 2b, 3c, and 4b.

b ND, not determined.

### Identification of end nucleotide in loop sequence connecting helix 73 and 74 for substrate activity by Erm protein.

In the study described above, a 7-nt hairpin loop sequence capping the methylatable adenine side of RNA substrate, which contains the whole sequence connecting helix 73 and 74 (**A**AAGA, bold nucleotide is the target adenine), could allow the resultant RNA fragment to be methylated by Erm proteins. Therefore, deletion was carried out starting from the 3′-end adenine in the loop sequence. Fortunately, because capping with the UUCG tetraloop on the other side could provide more efficient substrate activity, the hairpin loop could be unleashed to mimic the natural conformation and truncation of one nucleotide by one nucleotide was performed to define the end nucleotide, which allows methylation by the Erm protein to be facilitated. While the 24-nt RNA substrate containing all of the sequences (AAAGA) could be methylated by all of the Erm proteins, namely, ErmB, ErmE, and ErmS (Fig. 2b, Fig. 3c, d, and e), suggesting that 24 nt assumed the desired conformation (see below in Discussion), the 23-nt RNA fragment deprived of the 3′-end adenine residue could not be methylated by ErmB (Fig. 3b and d) and ErmE (Fig. 3b and e). In contrast to this, ErmS could display the methyl group-transferring activity toward this defective substrate. Beyond this point, until only the target adenine was left on the substrate, substrate capped with UUCG in place of A2051 could be methylated by ErmS without any loop sequence except the methylatable adenine (Fig. 3b and c). The loss of substrate activity could not be ascribed to the structural alteration by the deletion because deletions up to the target adenine could not abolish the methyl group-accepting activities by ErmS (see below in Discussion).

**FIG 3:**
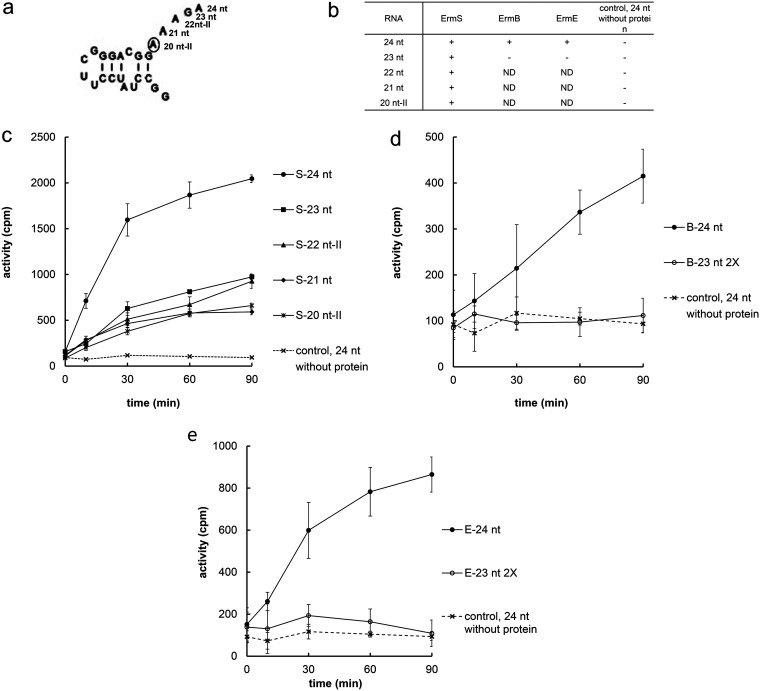
Determining the ultimate end on the side of the methylatable adenine for accepting a methyl group by Erm proteins. (a) The UUCG tetraloop in place of A2051 could support the methyl-transferring activity of all the Erm proteins employed in this study (ErmB, ErmE, and ErmS) to the substrate retaining unleashed AAAGA loop sequence connecting helix 73 to 74. The methylatable adenine is circled. (b) While ErmB and ErmE could not methylate the substrate (23 nt, −) in which just one nucleotide was truncated from 24 nt, ErmS could methylate all the substrates (+) including the one that retained the methylatable adenine alone. Substrate activity of all the substrates from 24 nt to 20 nt increased gradually with time by ErmS, confirming that the ultimate end is the methylatable adenine (c). For ErmB (d) and ErmE (e), no substrate activity increase could be observed over time just after one nucleotide was deleted from 24 nt. 2X denotes monitoring substrate activity with double the enzyme concentration.

### Methylation of substrate formed through annealing two strands displaying only a natural sequence by ErmS.

**FIG 4:**
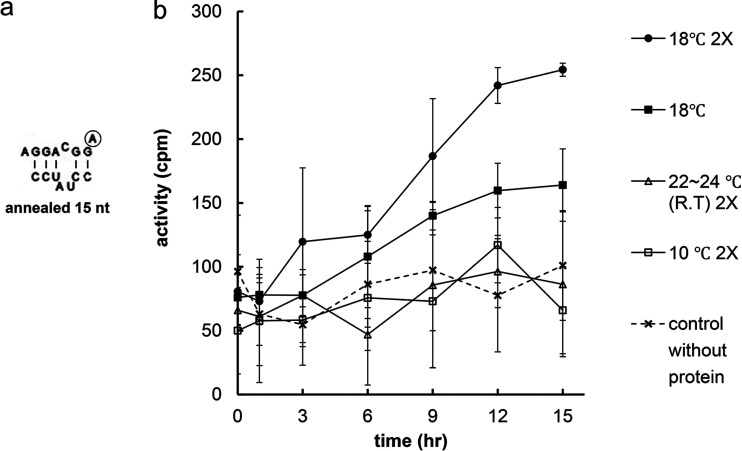
Methyl group-accepting activity of the substrate by ErmS, made up of two annealed RNA strands containing only a natural sequence. (a) Structure of annealed substrate (15 nt). Circled is the methylatable adenine. (b) Methylation was carried out with a substrate obtained by preincubating for 5 min at 50°C and annealing for 90 min at 10°C. Reaction mixture was incubated at the designated temperature in the presence of ErmS and SAM (refer to Results). At 18°C, the annealed substrate showed the methyl group-accepting activity with a concomitant increase of activity over time under both normal assay conditions and reaction conditions, where the levels of all of the components (enzyme, SAM, and substrate) were doubled (2X). However, at lower (10°C) and higher (24°C) temperatures alike, ErmS could not methylate it.

Artificially added sequences have been observed to affect substrate activity favorably, negatively, or not at all. To more precisely localize the minimal portion of 23S rRNA that could be recognized to be methylated by Erm proteins, an annealed construct of two strands containing only natural sequence was tested for its substrate activity with ErmS. The annealed construct was formed by bringing together the upper strand (AGGACGGA) and lower strand (CCUAUCC), resulting in 15-nt RNA. After annealing at 10°C, the duplex could not exhibit any methyl group-accepting activity with incubation at higher temperatures, such as 37°C and room temperature, even after 15 h of incubation. An incubation temperature as low as 18°C could allow the annealed duplex to display the methyl group-accepting activity, but when the incubation temperature decreased as low as the annealing temperature (10°C), substrate activity could not be supported. To confirm both the negative and the positive methylation activity by ErmS toward the annealed substrate, the levels of all reaction components, substrates, enzymes, and methyl group donors (SAM) were doubled, and the resultant transferred methyl group was detected with the conventional assay method (Fig. 4). With these assay conditions and incubation for 12 h at 18°C, an amount (0.002 pmol) of methyl group similar to that obtained with a pair of ErmE and 567 nt in the standard assay conditions could be transferred to the annealed substrate. Specific methylation of the annealed substrate at A2058 was confirmed with A2058U and A2058C mutant substrates (Table 1).

## DISCUSSION

It is already known that helix 73 and the loop sequence connecting helix 73 and 74 and containing the methylatable adenine are essential for the activity of all tested Erm proteins (9–12). Despite this observation, other structures in domain V have been demonstrated to function in recognition and methylation by Erm proteins (8, 20). It might be inferred that some portion of an essential part in helix 73 and the loop sequence could be deleted with the retention of substrate activity by the compensatory contribution of another part of domain V. However, in ErmS, such a compensatory effect by the large portion of the domain V structure beyond the methylatable adenine could not be observed upon the deletion of the A2051 nucleotide. It seemed that a compensatory effect works well with ErmE because the g21 nt substrate did not show any substrate activity with ErmE, whereas the 567-nt substrate could be methylated by ErmE, which has the same helix 73 end nucleotide of A2051 as the g21 nt substrate. However, when considering the conformation of the hairpin loop in g21 nt with restriction by tethering, this observation could not be explained clearly in this study. Although little or no compensatory effect was observed upon the deletion of nucleotides involved in essential protein-RNA interactions for the methylation by Erm proteins, truncation of the distal portion of helix 73 might instead suggest allostery and/or cooperativity between the distal region of helix 73 and the large structure of domain V beyond the target adenine in binding with the enzyme. Deletion of the terminal three base pairs induced an enormous reduction of substrate activity for all Erm proteins employed; only 16%, 30%, and 82% of the original activity of ErmB, ErmE, and ErmS was left after truncation. The observed huge decrease in substrate activity could not be ascribed to the role of just these three base pairs. Furthermore, a previous study (9) with ErmE indicated that in a 48-nt substrate containing only helix 73 and a hairpin loop harboring a sequence connecting helix 73 and 74, truncation of this distal region had no or little effect on substrate activity, possibly corroborating the allostery and/or cooperativity observed in this study. When truncation was carried out further up to A2051 to obtain 567-nt RNA, substrate activity was reduced to 1/356th and 1/147th of the activity of gg583 nt in ErmE and ErmS, respectively. For ErmB, with this truncation, substrate activity was completely abolished (for the reason on this, see below). Most of the decrease in substrate activity through the truncation of the terminal three base pairs could have been caused by allostery and/or cooperativity because they are located away from the essential binding site for methylation (minimal substrate region). However, the decrease in activity by the truncation from that point up to A2051 could have been caused by the loss of both allostery and/or cooperativity and supplementary binding affinity to that at the minimal substrate region, as could be observed in the case of the UUCG tetraloop (see below). In addition, individual differences in the tendency for decreasing substrate activity accompanying truncation of nucleotides in the distal region of helix 73 could also be observed because the extent of substrate activity loss by truncation varied depending on the Erm protein employed (Fig. 1a and b; refer to Results).

Even in the presence of the large portion of domain V beyond the methylatable adenine, the deletion of A2051 could abolish the substrate activity for ErmE and ErmS; the same phenomenon could also be observed with ErmS as well when a much smaller substrate, g21 nt, was used (Fig. 2b). Interestingly, when capped with the UUCG tetraloop in place of A2051 with the opposite loop region untethered (from 20 nt-I to 24 nt), substrate activity could be recovered substituting A2051 and was even enhanced with all the Erm proteins employed. With ErmS, 24 nt could exhibit more than four times higher substrate activity than the substrate equipped with A2051, g21 nt. With ErmE, this substrate (24 nt) exhibited three times higher substrate activity than 567 nt. The role of the UUCG tetraloop was conspicuous and surprising in the case of ErmB because it could not methylate even the substrates with A2051 (g21 nt and 567 nt), but the substrate capped with the UUCG tetraloop in place of A2051 (24 nt) exhibited methylation activity higher than that obtained with gg37 nt harboring the whole helix 73 sequence (1.3 times) (Fig. 2b). These observations, together with the fact that without A2051 ErmE and ErmS could not methylate the RNA substrate even in the presence of all other sequences beyond A2051, are notable and might suggest that affinity at or around A2051 is essential for the Erm protein to methylate 23S rRNA. Therefore, at least for ErmS, ErmB, and ErmE and presumably for all other Erm proteins, the ultimate end for methylation on the side furthest away from the methylatable adenine in helix 73 could be assigned to A2051 and/or proper binding affinity around position A2051. Using this quite good substrate (24 nt) with excellent substrate activity, further deletion of one more base pair next to A2051 toward the methylatable adenine side with retention of the UUCG cap did not lead us to obtain the methylatable substrate. This might mean that the base pair next to A2051 is essential for substrate activity and/or that the distance from the methylatable adenine to A2051 could be important for the substrate activity.

Once the ultimate end of the side furthest away from the methylatable adenine in helix 73 was designated, next, it would be necessary to define the ultimate end of the methylatable adenine side. Fortunately, the aforementioned efficient substrate (24 nt) that was capped with UUCG and equipped with an untethered AAAGA loop sequence could be used to determine the ultimate end of the methylatable adenine side (Fig. 3a). When just one nucleotide was removed from that substrate, substrate activity was completely lost with ErmB (Fig. 3b and d) and ErmE (Fig. 3b and e), as reported previously (9–12). For ErmB and ErmE, substrate activity can be observed when there is proper affinity around position A2051, such as that by the UUCG tetraloop, and all the structures and sequences from there on to the loop sequence connecting helix 73 and 74 are present. This is well congruent with the general belief that the loop sequence connecting helix 73 and 74 and containing the methylatable adenine is essential for all Erm protein activities, which seems reasonable because this loop sequence is located just next to the target adenine and possibly provides additional affinity to recognize and bind to the target adenine properly. However, surprisingly, ErmS retained the methylation activity with further truncation of the loop sequence until only the methylatable adenine remained. This observation, making further truncation impossible, enables us to conclude that this is the final and ultimate end for Erm protein methylation and that ErmS does not need additional binding on the 3′-end side for the recognition and proper binding of the target adenine (Fig. 3b and c). Further truncation inside the methylatable adenine might shorten the distance between the two ultimate ends of the methylatable adenine and the position of A2051, not allowing the essential interaction and abolishing the substrate activity, as observed above. Another specific feature could be encountered with Erm proteins in methylating the minimal substrate besides that observed above. While ErmS requires only the target adenine for its methylation activity if the sequence from A2051 to the GC base pair just before the methylatable adenine is provided, an additional four nucleotides in the connecting loop between helix 73 and 74 (**A**AAGA, bold A denoting the methylatable adenine) after the target adenine are necessary for the methylation activity by ErmE. In addition, for ErmB to exhibit the methylation activity, stronger binding affinity might be necessary at or around the position of A2051, in addition to the sequence required by ErmE. To remove any effect from an unnatural sequence, two oligonucleotides containing just the natural sequence were annealed and assayed using ErmS, and substrate activity could be detected at temperatures as low as 18°C. Even at room temperature, no substrate activity could be detected, presumably due to the instability of the duplex. At another temperature tested, 10°C, ErmS could not exhibit methylation activity, possibly due to this temperature being too low for proper enzymatic activity. Now, the plausible smallest portion of domain V, a complete substrate for Erm protein, could be defined to be bound, recognized, and methylated by the Erm protein, which was well congruent with the observations obtained for each ultimate end of substrate. This portion was a 15-nt irregular duplex followed by the target adenine, presumably a similar structure to that of 23S rRNA from A2051 to the methylatable adenine.

From this study, it is apparent that ErmS could methylate A2058, recognizing and binding the smallest portion of 23S rRNA. To date, most studies on Erm proteins have been based on the premise that these proteins function quite similarly, with the common goal of protecting cells from the inhibitory action of antibiotics. Proteins are supposed to change and evolve to adapt to the needs posed by stimuli from the environment. Each member of the Erm protein family might methylate the target adenine located in a highly conserved region, the peptidyl transferase loop, in a similar manner. However, from the details of methylation, such as the recognition of the minimal part of domain V for methylation and distinct decreases in substrate activity upon the same deletion as described in this study, distinct and specific features could be observed among the Erm proteins. This is reasonable because even the peptidyl transferase loop, which contains a highly conserved sequence and structure, could harbor some variations; this is also true for Erm proteins, despite them being grouped as a family. Another aspect for distinguishing Erm proteins from each other could be their origin, namely, pathogen (ErmB) or antibiotic producer (ErmS and ErmE). ErmS functions to avoid the suicidal effects of the endogenous antibiotic tylosin, with the other three resistance proteins (21–24) in Streptomyces fradiae, a tylosin producer, and ErmE acting as a sole resistance factor protein in the antibiotic erythromycin producer Saccharopolyspora erythraea (25–27). In contrast to these proteins, Erm proteins, such as ErmB, residing in pathogens would work only at limited times when one or more exogenous antibiotics were present with pathogens. According to our study, ErmS might require and methylate the smallest part of domain V, a complete substrate of Erm proteins, which is possibly one of the most equipped Erm proteins in methylating substrate RNA. That is, it probably might show how nature can methylate the substrate by the Erm protein recognizing and interacting with an RNA substrate that is as small as possible.

The 24-nt RNA, which was equipped with a UUCG tetraloop and one of the two substrates (with g21 nt) at the pivotal position in developing minimal substrate, presumably has the secondary structure shown in Fig. 2a and 3a for the following reasons. The UUCG tetraloop could provide exceptional stability for 24 nt RNA with the help of a closing base pair (G2052:C2617) presented by helix 73, and the resulting C(UUCG)G tetraloop could help in the folding of 24-nt RNA by initiating the folding process as a nucleation site, facilitating formation of an intramolecular double-stranded RNA helix (28, 29). The stability of the UUCG tetraloop hairpin structure could be maintained without any preferences for the sequence and length of the stem, while shorter stems were observed to be closed more frequently by the UUCG tetraloop, probably due to its higher stability (30). Therefore, once the G2052:C2617 base pair and the nearby UUCG tetraloop form a tetraloop structure unit, remaining base pairs could be easily fitted in the folding process until it met the nucleotide(s) which cannot base pair with any other nucleotide. C2055 (in E. coli coordinate) and A2164 likely exist as unpaired nucleotides because they could not find any possible base-pairing partner. In contrast, U2163 could form a base pair with G2056 without adding any nucleotide into the internal loop formed by C2055/A2164. After that, the G2057:C2612 base pair could form, leaving C2611 unpaired along with artificially added GG dinucleotides for successful *in vitro* transcription. Alternatively, U2163 exists as a member of the internal loop which could be closed by the two base pairs G2056:C2612 and G2057:C2611, as observed in the secondary structure derived by phylogenetic sequence comparison (17, 18) and reflected in the functional ribosomal particles (31), as presented in this study. Moreover, single-stranded **A**AAGA (**A** denotes the target adenine) should follow two structures being unpaired. These two proposed structures were predicted by Mfold (42) as the most probable secondary structures at the condition of 1 M NaCl and 37°C. However, in one of them, the bottom strand internal loop size is reduced from two to one. This reduction of size had been reported to decrease the substrate activity to two-thirds of the original activity of domain V with ErmE (19). This decrease in the substrate activity by this modification could be exacerbated in the minimal substrate structure, such as 41 nt, with only 3% of substrate activity left after the size reduction with ErmS (12; and H.J.L. and H.J.J., unpublished results). Therefore, the 24-nt RNA substrate prepared in this study likely has the secondary structure maintaining C2055/U2613 and A2614 as internal loop as shown in Fig. 2a and 3a because it exhibited reasonably high substrate activity with all three Erm proteins tested (Fig. 2b). The 3′-dangling-end nucleobase(s) in RNA would stabilize the neighboring duplex by protecting the closing base pair from the solvent through synergy between the hydrogen bond stabilization in the closing base pair and favorable stacking interaction (32). In the 3′ end of 24-nt RNA, the single-stranded adenine-rich overhang **A**AAGA (**A**, the methylatable adenine) may have the A-type single helical stacking conformation which reduces the dynamics of the innermost dangling base through nearest-neighbor intrastrand stacking interactions with the geometry that will not hamper the methyl group accepting activity. This preorganized and relatively more rigid single-stranded helical overhang provides a stronger dangling-end stabilization effect to the closing base pair of the RNA duplex than the single dangling nucleobase (32–34). Therefore, deletion of nucleotide(s) from this sequence may destabilize the helix structure of 24-nt RNA and may destroy the substrate activity by reducing the length of the preorganized single-stranded region and its stabilizing effect. However, the methyl group-accepting activity with ErmS could be maintained until only the methylatable adenine was left by the nucleotide-wise truncation from 23 nt to 20 nt-II, while deletion of A2062 alone could abolish the substrate activity of the resultant 23 nt with ErmB and ErmE. This observation suggests that in the minimal 20-nt substrate, the appropriate conformation is retained to accept the methyl group transferred by ErmS. Therefore, structural perturbation by deletion could not be the cause of the complete loss of methyl group-accepting activity with ErmB or ErmE.

The transition from 24-nt RNA to g21 nt RNA reduced substrate activity to be one-fourth as much activity. While tetraloops, such as UNCG, GNRA, and CUYG, could provide exceptional stability to the resultant hairpin structure, hairpin loops with six to seven unpaired nucleotides have been also known to bring optimal stability to the hairpin structure through maximizing stacking interactions and satisfying steric constraints imposed by the following helix structure (35). However, unlike UUCG in 24-nt RNA, it is not easy to designate base pair(s) working as an nucleation core even though one or more GC base pairs or the likes of those in the supposed helix would take that role. Therefore, the size of the loop and the helix irregularity could be the major determinants of the final structure of the hairpin in postulating the structure of the minimized RNA substrate. As described above, a 7-nt capping loop, composed of a natural single-stranded sequence (**A**AAGA, bold letter denotes the methylatable adenine) and artificially added CC after that, has been used in previous reports to determine the substrate activity of several minimal RNAs encompassing various lengths of the helix 73 sequence, presumably taking advantage of the stabilizing property of the 7-nt loop for the following helical structure, as could be observed in many tRNA structures. This 7-nt loop structure was observed to successfully support the proposed secondary structures, even though those structures harbored more base pairs forming the helix structure on the other side of loop than the minimal substrates employed in this study (by three base pairs in the case of the smallest substrate), which might help to maintain the proposed structure (9, 10). If artificially tethered nucleotides for the formation of the hairpin exist as planned, a 7-nt loop, the resultant structure, should assume the natural conformation, leaving added G for successful *in vitro* transcription and A2051 unpaired, as shown in Fig. 2a. Haasnoot et al. (35) proposed that in RNA, six to seven-membered loops could be the most stable. If a capping sequence formed the loop comprising six-nucleotide residues (**A**AAGAC, **A** denotes the target adenine), one more C residue (2611) was added to the bottom strand of the internal loop (total three nucleotides) while maintaining the same 5′ end of dangling G and A(2051) and helix structure as the natural one might adopt. This substrate will likely lose the substrate activity due to steric hindrance by the increased loop size, presumably because of the stringency of the requirement of a 3-nucleotide internal loop (see below). Furthermore, Haasnoot et al. suggested that longer and shorter lengths of a hairpin loop were possible alternatives. Mfold predicted the structures with these different lengths of loop sequences from 5 to 8 nucleotides for the g21-nt RNA substrate. With the 5-nt loop (**A**AAGA), the internal loop changed to a two-residue bulge (A/C2054/2055) in the upper strand without any unpaired nucleotide in the bottom strand. This modification may destroy the substrate activity of the minimal substrate by depriving the bottom strand unpaired sequences, as even truncation of only one nucleotide could take away almost all of the substrate activity in the context of minimal substrate structure. Even though it retains some substrate activity, it could be hardly detected (see below). Another possibility is an eight-membered loop structure (**A**AAGACCC) including C2611 in the hairpin loop sequence. With this loop, the substrate in which the bottom strand internal loop size was reduced from two to one, as seen previously in 24-nt RNA. Following the same reasoning, this substrate could lose most of the methyl group-accepting activity. Therefore, A2051 along with artificially added G likely exists as a dangling end to the part of helix 73 in the minimal substrate with the 7-nt loop, retaining the substrate activity. Even though it has been known to show a directional difference in dangling end stabilization and 5′ dangling has a much lower stabilization effect on the RNA duplex than the 3′-end overhang, the 5′ dangling end, such as A2051, could exert structural stabilization on g21 nt (Fig. 2a). Therefore, deletion of A2051 could induce structural disruption and cause the loss of the substrate activity. To test this hypothesis in the g21-nt substrate, A2051C and A2051U mutations were introduced and their substrate activities were analyzed. As seen in Fig. 2b and c, no methyl group-accepting activity could be observed when mutant substrates were used, while wild-type g21 nt clearly exhibited substrate activity, as expected. This observation suggests an interaction between ErmS and A2051. Deletion of A2051 caused no structural disruption, leading to the loss of the substrate activity, corroborating the observation that substrate activity could be enhanced with a substrate equipped with a UUCG tetraloop for all the Erm proteins tested in this study and the notion that affinity between the Erm protein and RNA substrate at or around A2051 is essential for substrate activity, at least in the context of minimal substrates.

It is to be expected that the same 7-nt loop sequence was adopted for determining the substrate activity of a variety of constructs harboring the helix 73 sequence in this study because, in the previous studies, this loop has been shown to successfully support the RNA substrate in retaining the natural substrate structures and activities, encompassing various lengths of helix 73 (9, 10, 12). A shorter sequence in helix 73 than those used in previous studies might not have the same effect. Again, for minimal RNA substrates, a three-nucleotide internal loop structure plays critical role(s) for the substrate activity because disruption of that structure almost completely abolished the methyl group-accepting activity, especially in the context of a minimal RNA substrate. The previous reports (12, 19) allowed us to peek into what could happen here. In domain V, a complete substrate for Erm proteins, deletion of C2055 could reduce the methyl group-accepting activity by 80% and changing of the internal loop structure to base pair could also lead to a similar result (80% reduction of substrate activity) with ErmE. Activity loss with a disturbance of the internal loop worsened in the minimal substrate, such as the 41-nt RNA substrate which contains the whole helix 73; with deletion of C2055, less than 0.1% activity remained; with deletion of U2013, 3% remained; and truncation of A2014 resulted in 0.2% of wild-type activity. The internal loop structure and its sequence were almost 100% conserved in bacteria. In addition, Erm proteins exist because they could save microorganisms from the action of antibiotics in the MLS_B_ antibiotic superfamily. Erm proteins recognize not only sequence motifs (primary structure) but also structure motifs (secondary and/or higher structural characteristics), presumably to maintain the fidelity and specificity of methylation without any compromise of substrate specificity. If Erm proteins had rather broad structural specificity, their methyltransferase activity would leak unnecessarily and thus could not maintain the biological economy in the “life or death” situation. The target adenine is located in the region containing a peptidyl transferase loop which is formed at the junction of five helices that are linked together by phylogenetically highly conserved single-stranded regions and is thus assumed to fold into a nearly identical structure in different organisms. In accordance with this, the ErmE from Saccharopolyspora erythraea and the ErmS from Streptomyces fradiae and other Erm proteins could modify rRNA from diverse Gram-positive and -negative bacteria (36, 37). Furthermore, as described above, there has been a consensus that some length of helix 73 attached to the methylatable adenine is essential for the methylating activity of Erm proteins on the minimal substrate, suggesting the integrity of the secondary structural features could be prerequisite for proper methylation. While larger substrates, such as domain V, could be methylated by Erm proteins without relying on the structures provided by minimal substrates, maintaining the secondary structure might be essential for the activity of the minimal substrates, especially if the primary structure is provided as in the natural sequence. The substrate activity was reduced by three-fourths by the transition from 24 nt to g21 nt, and g21 nt could be considered to exhibit a reasonably high substrate activity when taking into account the enhanced activity by the additional affinity provided by the UUCG tetraloop. Therefore, this observation has ruled out the possibility that shorter helix sequences than those used in the previous studies compromise the proper secondary structure. Furthermore, two strands of natural sequence were annealed properly and showed substrate activity, suggesting that it has assumed the proper structure even though structural instability could be observed due to a lack of hairpin loop structure for its structural stabilization. This observation might again corroborate the hypothesis that g21-nt and 24-nt substrates have a natural conformation, as observed in Fig. 2a in this study and the previous reports. Furthermore, g21 ntA2051U and g21 ntA2051C lost its substrate activity, as did 20 nt-I, suggesting that a transition from g21 nt to 20 nt-I by the deletion of A2051 did not induce any structural perturbations. Following the same line of reasoning, 567-nt and 566-nt RNA substrates might assume a similar secondary structure in the helix 73 region, as could be observed in the case of g21-nt, 24-nt, and 20-nt-I RNA. Besides harboring the same part of the helix 73 sequence as g21 nt, 567 nt is deprived of the potential for cooperativity and/or allostery between the region further above A2051 from the target adenine in helix 73 and the large structure beyond the methylatable adenine in domain V, and so much of its substrate activity should depend on the minimal region of helix 73 and its secondary structure. However, while 567 nt exhibited high substrate activity, 566 nt and 567 ntA2051U and 567 ntA2051C mutants lost the methyl group-accepting activities simultaneously, suggesting that as in g21 nt, deletion of A2051 did not induce any structural disturbance with the loss of the substrate activity.

## MATERIALS AND METHODS

### Materials.

E. coli DH5a (Promega, Madison, WI, USA) and BL21(DE3) (Novagen, Madison, WI, USA) were used for general cloning and expression of His6-tagged Erm proteins, respectively. The sequence for domain V of 23S rRNA used in this study was cloned from Bacillus subtilis BD170 and exhibited three sequence differences compared with the one presented on the Gutell Lab’s Comparative RNA Website (CRW site; http://www.rna.icmb.utexas.edu), including two mutations (C2203G and U2629A) and one nucleotide deletion (Δ C2473). While 19 identical sequences were identified upon a search of GenBank with our sequence as a query, only 2 sequences showed exact sequence identity with the one from the Gutell Lab’s CRW site. Restriction endonucleases and DNA-modifying enzymes were purchased from New England BioLabs (Beverly, MA, USA) and used as recommended by the supplier’s manual. LB media and Bacto agar for bacterial culture were from Difco Laboratories (Detroit, MI, USA). For PCR, *Taq* polymerase and nucleotides were obtained from TaKaRa Shuzo Co. (Otsu, Shiga, Japan). For *in vitro* transcription, spermine, Triton X-100, and polyethylene glycol (PEG; molecular weight, 8,000) were obtained from Sigma Chemical Co. (St. Louis, MO, USA), and nucleotides were obtained from TaKaRa Shuzo Co. The T7 RNA polymerase was prepared “in-house.” The chemically synthesized ribooligonucleotides were purchased from Bioneer Co. (Daejeon, South Korea). The His·Bind resin was from Novagen. Reagents for polyacrylamide gel electrophoresis, such as acrylamide, bis-acrylamide, ammonium persulfate, and *N,N,N*′,*N*′-tetramethylethylenediamine (TEMED), were obtained from Bio-Rad (Hercules, CA, USA). Most of the conventional chemicals, such as salts, buffer components, agarose, and antibiotics, were purchased from Sigma Chemical Co.

### Construction of expression vector.

The expression vector (pHJJ105) and E. coli strain (E. coli HJJ105) overexpressing ErmS and those (pHJJ202 and E. coli HJJ202) for ErmE were obtained in previous studies (15, 20). For constructing an ErmB expression vector, pVA838 plasmid DNA (38) was used as a DNA template to obtain the mature protein-coding region. Two oligonucleotides, 5′-GGAATTCcatatgAACAAAAACATCAAA TACTCTCAAAACTTTTTAACGAAT-3′ and 5′-CCGctcgag TTTCCTCCCGTTAAATAATAG-3′, were used as forward and reverse primers, respectively, for the amplification of *ermB* by PCR. The sequences of primers were modified to include a restriction site for NdeI (5′-catatg-3′), overlapping the initiation Met codon, and a site for Xhol (5′-ctcgag-3′). The resultant PCR product was directly digested with NdeI and Xhol restriction enzymes, and the DNA fragment containing the *ermB* gene was ligated into pET23b NdeI-Xhol sites. The resulting plasmids were designated pHJJ302. The cloned gene was sequenced to confirm the sequence and frame of the insert.

### Protein expression and purification.

Each methyltransferase was expressed from E. coli BL21(DE3) harboring plasmid pHJJ105 (ErmS), pHJJ202 (ErmE), and pHJJ302 (ErmB). Purification was performed by a slightly modified version of a previously described procedure (20). Briefly, cells from 100 ml of culture were collected by centrifugation and resuspended in buffer A (20 mM Tris-HCl [pH 7.0], 500 mM KCl, and 5 mM imidazole). The cells were disrupted by sonication on ice using a GEX-130 ultrasonic processor (130 W, 20 kHz) at 50% amplitude for 5-s pulses with 10-s pauses for cooling. The total sonication time was 5 min. The lysate was centrifuged to remove the cell debris and other insoluble materials, including inclusion bodies, and the supernatant was loaded onto a column containing His·Bind resin preequilibrated with buffer A. Next, the column was washed extensively with buffer B (20 mM Tris-HCl [pH 7.0], 500 mM KCl, and 100 mM imidazole) to remove unbound and falsely bound proteins, and proteins were eluted with buffer C (20 mM Tris-HCl [pH 7.0], 500 mM KCl, and 300 mM imidazole). To remove the imidazole and salt, the eluted protein solution was purified using a PD-10 desalting column, as described by GE Healthcare (Little Chalfont, Buckinghamshire, UK) and was stored at −20°C in 20 mM Tris-HCl (pH 7.0), 200 mM KCl, 1 mM EDTA, and 50% glycerol. The protein concentration was determined by the bicinchoninic acid (BCA) protein assay method (Pierce, Rockford, IL, USA).

### Cloning for *in vitro* transcription of various rRNAs.

For the production of transcripts with homogeneous 3′ ends, the hepatitis delta virus (HDV) ribozyme was incorporated into the 3′ end of a target RNA (39), of which constructs were generated by a series of PCRs. To obtain the DNA fragment encoding gg583 nt, the first PCR was performed using B. subtilis BD170 chromosomal DNA as the template and oligonucleotides oligo-2 (an appropriate oligo-2 derivative was employed for each A2058 mutant) and oligo-3 as forward and reverse primers containing part of the T7 promoter and 15 nt of the 3′-end sequence of HDV, respectively. Parts of the sequences of these primers correspond to nucleotides 2043 to 2063 and 2606 to 2625 (E. coli coordinates; 2070 to 2090 and 2634 to 2653 in B. subtilis coordinates) in B. subtilis 23S rRNA. The EcoRI restriction site, a remaining partial phage T7 promoter sequence to make it complete (in oligo-1 in Table 2), and an additional HDV ribozyme sequence along with an overlapping sequence (bold and italicized letters of oligo-3 and oligo-4 in Table 2) were introduced during the second PCR, which employed oligo-1 as a forward primer and oligo-4 as a reverse primer. To introduce the remnant HDV ribozyme sequence to complete it and the XbaI restriction site, the third PCR was performed using the second PCR product DNA as the template and oligo-1 and oligo-5 as forward and reverse primers, respectively. After amplification, the DNA fragment encoding gg583 nt DNA was cloned into pUC19 using EcoRI and XbaI, and sequencing was performed to confirm the sequence. DNA of 577 nt (nucleotides 2046 to 2622 in E. coli coordinates, oligos-6 and -7), 567 nt (2051 to 2617, oligos-8 and -10; for each mutant of A2051 and A2058, an appropriate oligo-8 derivative was used), and 566 nt (2052 to 2617, oligos-9 and -10) was also cloned in the same manner as described above. But, when performing the second and third PCR for 567 nt, oligo-1-1 was employed instead of oligo-1 because it starts with adenosine.

**TABLE 2:** DNA oligonucleotides used in cloning DNA fragments encoding various rRNA constructs

DNA oligonucleotide	Sequence (5′–3′)	Description
Oligo-1	G**GAATTC**taatacgactcactataG	25-mer forward primer for rRNA DNA cloning, containing T7 promoter sequence (lowercase) and restriction enzyme site (EcoRI, bold)
Oligo-1-1	G**GAATTC**taatacgactcactata	24-mer forward primer for 567-nt DNA cloning, containing T7 promoter sequence (lowercase) and restriction enzyme site (EcoRI, bold)
Oligo-2	cgactcactataGGCCCGCGACAGGACGGAAAGAC	35-mer forward primer for gg583-nt DNA cloning, containing pT7ps (partial T7 promoter sequence, lowercase)
Oligo-2-1	cgactcactataGGCCCGCGACAGGACGG**G**AAGAC	35-mer forward primer for A2058G mutant gg583-nt DNA cloning, containing pT7ps (partial T7 promoter sequence, lowercase; A2058G mutated nucleotide, bold)
Oligo-2-2	cgactcactataGGCCCGCGACAGGACGG**C**AAGAC	35-mer forward primer for A2058C mutant gg583-nt DNA cloning, containing pT7ps (partial T7 promoter sequence, lowercase; A2058C mutated nucleotide, bold)
Oligo-2-3	cgactcactataGGCCCGCGACAGGACGG**T**AAGAC	35-mer forward primer for A2058U mutant gg583-nt DNA cloning, containing pT7ps (partial T7 promoter sequence, lowercase; A2058U mutated nucleotide, bold)
Oligo-3	***GGGACCATGGCCGGC***CCCGCGACGGATAGGGACCG	35-mer reverse primer for gg583-nt DNA cloning, containing 15 nt 3′-end HDV ribozyme sequence (bold and italicized)
Oligo-4	***GTCCCCTCGGAATG****TTGCCCACCGGCCGCCAGCGAGGAGGCT****GGGACCATGGCCGGC***	57-mer reverse primer for rRNA DNA cloning, covering pHDVrs (partial HDV ribozyme sequence, italicized), some of which overlapped with oligos-3 (bold and italicized) and -5 (bold, italicized, and underlined) to add more HDV ribozyme sequence
Oligo-5	GCTCTAGA*GTCCCATTCGCCATTACCGAGGGGACG****GTCCCCTCGGAATG***	49-mer reverse primer for rRNA DNA cloning, containing pHDVrs (italicized), some of which overlapped with oligo-4 (bold, italicized, and underlined) and restriction enzyme site (XbaI, underlined)
Oligo-6	cgactcactataGCGACAGGACGGAAAGACCCC	33-mer forward primer for 577-nt DNA cloning, containing pT7ps
Oligo-7	***GGGACCATGGCCGGC***GCGACGGATAGGGACCGAAC	35-mer reverse primer for 577-nt DNA cloning, containing 15-nt 3′-end HDV ribozyme sequence (bold and italicized)
Oligo-8	cgactcactataAGGACGGAAAGACCCCGTGG	32-mer forward primer for 567-nt DNA cloning, containing pT7ps
Oligo-8-1	cgactcactataAGGACGG**G**AAGACCCCGTGG	32-mer forward primer for 567-nt DNA cloning, containing pT7ps; A2058G mutated nucleotide, bold
Oligo-8-2	cgactcactataAGGACGG**C**AAGACCCCGTGG	32-mer forward primer for 567-nt DNA cloning, containing pT7ps; A2058C mutated nucleotide, bold
Oligo-8-3	cgactcactataAGGACGG**T**AAGACCCCGTGG	32-mer forward primer for 567-nt DNA cloning, containing pT7ps; A2058U mutated nucleotide, bold
Oligo-8-4	cgactcactata**C**GGACGGAAAGACCCCGTGG	32-mer forward primer for A2051C mutant 567-nt DNA cloning, containing pT7ps; A2051C mutated nucleotide, bold
Oligo-8-5	cgactcactata**T**GGACGGAAAGACCCCGTGG	32-mer forward primer for A2051U mutant 567-nt DNA cloning, containing pT7ps; A2051U mutated nucleotide, bold
Oligo-9	cgactcactataGGACGGAAAGACCCCGTGGAG	33-mer forward primer for 566-nt DNA cloning, containing pT7ps
Oligo-10	***GGGACCATGGCCGGC***GGATAGGGACCGAAC	30-mer reverse primer for 567/566-nt DNA cloning, containing 15-nt 3′-end HDV ribozyme sequence, bold and italicized
Oligo-11	cgactcactataGGCCCGCGACAGGA**CGGAAAGACCCCTATCCGTCGCG**	49-mer forward primer for gg37-nt DNA cloning, containing pT7ps (lowercase) and sequence complementary to oligo-12 (23 nt, bold)
Oligo-12	***GGGACCATGGCCGGC***CC**CGCGACGGATAGGGGTCTTTCCG**	40-mer reverse primer for gg37-nt DNA cloning, containing 15-nt 3′-end HDV ribozyme sequence (bold and italicized) and sequence complementary to oligo-11 (23 nt, bold)
Oligo-13	cgactcactataGAGGACGGAAAGACCCCTATCC***GCCGGCCATGGTCCC***	49-mer forward primer for g21-nt DNA cloning, containing pT7ps (lowercase) and pHDVrs overlapping with 15-nt 3′-end sequence of oligo-4 (both are italicized and bold)
Oligo-13-1	cgactcactataGAGGACGG**G**AAGACCCCTATCC***GCCGGCCATGGTCCC***	49-mer forward primer for A2058G mutant g21-nt DNA cloning; A2058G mutated nucleotide, bold
Oligo-13-2	cgactcactataGAGGACGG**C**AAGACCCCTATCC***GCCGGCCATGGTCCC***	49-mer forward primer for A2058C mutant g21-nt DNA cloning; A2058C mutated nucleotide, bold
Oligo-13-3	cgactcactataGAGGACGG**T**AAGACCCCTATCC***GCCGGCCATGGTCCC***	49-mer forward primer for A2058U mutant g21-nt DNA cloning; A2058U mutated nucleotide, bold
Oligo-13-4	cgactcactataG**C**GGACGGAAAGACCCCTATCC***GCCGGCCATGGTCCC***	49-mer forward primer for A2051C mutant g21-nt DNA cloning; A2051C mutated nucleotide, bold
Oligo-13-5	cgactcactataG**T**GGACGGAAAGACCCCTATCC***GCCGGCCATGGTCCC***	49-mer forward primer for A2051U mutant g21-nt DNA cloning; A2051U mutated nucleotide, bold
Oligo-14	cgactcactataGGACGGAAAGACCCCTATCC***GCCGGCCATGGTCCC***	47-mer forward primer for 20-nt-I DNA cloning, containing pT7ps (lowercase) and pHDVrs overlapping with 15-nt 3′-end sequence of oligo-4 (both are italicized and bold)
Oligo-15	cgactcactataGGCCTATCC**ttcg**GGACGGAAAGA***GCCGGCCATGGTCCC***	51-mer forward primer for 24-nt DNA cloning, containing pT7ps (lowercase) and pHDVrs overlapping with 15-nt 3′-end sequence of oligo-4 (both are italicized and bold); Ttcg denotes UUCG tetraloop after *in vitro* transcription
Oligo-15-1	cgactcactataGGCCTATCC**ttcg**GGACGG**G**AAGA***GCCGGCCATGGTCCC***	51-mer forward primer for A2058G mutant 24-nt DNA cloning; A2058G mutated nucleotide, bold
Oligo-15-2	cgactcactataGGCCTATCC**ttcg**GGACGG**C**AAGA***GCCGGCCATGGTCCC***	51-mer forward primer for A2058C mutant 24-nt DNA cloning; A2058C mutated nucleotide, bold
Oligo-15-3	cgactcactataGGCCTATCC**ttcg**GGACGG**T**AAGA***GCCGGCCATGGTCCC***	51-mer forward primer for A2058U mutant 24-nt DNA cloning; A2058U mutated nucleotide, bold
Oligo-16	cgactcactataGGCCTATC**ttcg**GACGGAAAGA***GCCGGCCATGGTCCC***	49-mer forward primer for 22-nt-I DNA cloning, containing pT7ps (lowercase) and pHDVrs overlapping with 15-nt 3′-end sequence of oligo-4 (both are italicized and bold); Ttcg denotes UUCG tetraloop after *in vitro* transcription
Oligo-17	cgactcactataGGCCTATCC**ttcg**GGACGGAAAG***GCCGGCCATGGTCCC***	50-mer forward primer for 23-nt DNA cloning, containing pT7ps (lowercase) and pHDVrs overlapping with 15-nt 3′-end sequence of oligo-4 (both are italicized and bold); Ttcg denotes UUCG tetraloop after *in vitro* transcription
Oligo-18	cgactcactataGGCCTATCC**ttcg**GGACGGAAA***GCCGGCCATGGTCCC***	49-mer forward primer for 22-nt-II DNA cloning, containing pT7ps (lowercase) and pHDVrs overlapping with 15-nt 3′-end sequence of oligo-4 (both are italicized and bold); Ttcg denotes UUCG tetraloop after *in vitro* transcription
Oligo-19	cgactcactataGGCCTATCC**ttcg**GGACGGAA***GCCGGCCATGGTCCC***	48-mer forward primer for 21-nt DNA cloning, containing pT7ps (lowercase) and pHDVrs overlapping with 15-nt 3′-end sequence of oligo-4 (both are italicized and bold); Ttcg denotes UUCG tetraloop after *in vitro* transcription
Oligo-20	cgactcactataGGCCTATCC**ttcg**GGACGGA***GCCGGCCATGGTCCC***	48-mer forward primer for 20-nt-II DNA cloning, containing pT7ps (lowercase) and pHDVrs overlapping with 15-nt 3′-end sequence of oligo-4 (both are italicized and bold); Ttcg denotes UUCG tetraloop after *in vitro* transcription
Oligo-20-1	cgactcactataGGCCTATCC**ttcg**GGACGG**G*GCCGGCCATGGTCCC***	48-mer forward primer for A2058G mutant 20-nt-II DNA cloning; A2058G mutated nucleotide, bold
Oligo-20-2	cgactcactataGGCCTATCC**ttcg**GGACGG**C*GCCGGCCATGGTCCC***	48-mer forward primer for A2058C mutant 20-nt-II DNA cloning; A2058C mutated nucleotide, bold
Oligo-20-3	cgactcactataGGCCTATCC**ttcg**GGACGG**T*GCCGGCCATGGTCCC***	48-mer forward primer for A2058U mutant 20-nt-II DNA cloning; A2058U mutated nucleotide, bold

Shorter DNA fragments for *in vitro* transcription of substrate RNAs were generated by splicing with a slightly modified version of the overlap extension method (40). DNA fragments encoding gg37 nt RNA were generated by a series of PCRs to include the HDV ribozyme at the 3′ end as well. The first PCR was performed with oligos-11 and -12, containing complementary sequences to each other and covering the whole gg37 nt RNA sequence, which could be generated by combining the sequences from both oligonucleotides. The second (using oligo-1 and oligo-4 as forward and reverse primers) and third PCRs (using oligo-1 and oligo-4 as forward and reverse primers) were carried out by the procedure described above. The DNA fragments encoding g21 nt, 20 nt-I, 24 nt, 22 nt-I, 23 nt, 22 nt-II, 21 nt, and 20 nt-II RNA were generated by PCR with oligos-13 to -20 as a forward primer, respectively, and oligo-4 as a reverse primer, which contained complementary sequences to each other. To generate the mutants for A2051 and A2058 of 24 nt, g21 nt and 20 nt-II, an appropriate forward primer in Table 2 was adopted for first PCR. To include the EcoRI restriction site and make the T7 promoter sequence complete at the 5′ end, as well as to finish the HDV ribozyme sequence and add the XbaI restriction site at the 3′ end, the final PCR employing oligo-1 and oligo-5 as forward and reverse primers, respectively, was carried out in the presence of each DNA fragment of the eight different constructs obtained above as the template. All the resultant DNA fragments were inserted into the multicloning sites of EcoRI and XbaI in pUC19. The absence of unwanted mutations was confirmed by DNA sequencing. All primers for the cloning of rRNA-encoding DNA fragments are summarized in Table 2.

### *In vitro* transcription of substrate RNAs.

RNAs were all transcribed *in vitro* using phage T7 RNA polymerase. The plasmids constructed and described above were used as the templates for *in vitro* transcription. Plasmids were linearized with XbaI for runoff transcription. The linearized plasmids were used directly as the template for the synthesis of substrate RNA transcripts. Transcription from the linearized plasmid templates was performed in 500-μl reaction mixtures containing 40 mM Tris-HCl (pH 8.1), 5 mM dithiothreitol (DTT), 1 mM spermine, 0.01% Triton X-100, 80 mg/ml PEG, 25 μg of DNA template, 4 mM ribonucleoside triphosphates (rNTPs), 28 mM MgCl_2_, and 10 μg of T7 RNA polymerase (prepared in-house) at 37°C for 4 h. After transcription, the transcripts were extracted with phenol-chloroform, precipitated with ethanol, and resuspended in diethyl pyrocarbonate (DEPC)-treated water. Following transcription, the concentration of MgCl_2_ was adjusted to 40 mM, and it was subjected to three rounds of thermal cycling (1 min at 72°C, 5 min at 65°C, and 10 min at 37°C per round) to carry out the self-cleavage reaction of 3′ HDV ribozyme (39). The transcripts produced were visualized with UV on a 5% to 13% 7 M urea-polyacrylamide gel to check the integrity and verify the size. The band with the correct size was extracted in a Tris-borate-EDTA (TBE) buffer by electrophoresis; and after ethanol precipitation, it was dissolved in self-folding buffer (50 mM HEPES-KOH [pH 7.5], 20 mM magnesium acetate, and 400 mM NH_4_Cl), heated to 65°C for 10 min, and then cooled to 37°C over a period of 90 min to complete the self-folding of RNA transcripts.

### *In vitro* methylation assay.

*In vitro* methylation of RNA substrates by Erm proteins was carried out by a slightly modified version of a previously described procedure (6, 8, 20, 41). For the time course analysis, reactions were performed in 300-μl volumes containing 50 mM Tris-HCl (pH 7.5), 4 mM MgCl_2_, 40 mM KCl, 10 mM dithiothreitol, 19.8 pmol *S*-adenosyl-l-methionine (SAM; specific activity, 80 Ci/mmol; PerkinElmer), 60 pmol rRNA transcripts, and 60 pmol purified Erm proteins; the volume and components were sufficient for six reactions. Reaction mixtures containing everything except proteins were prewarmed to 37°C by at least 5 min of incubation, and then purified Erm proteins were added to prewarmed tubes to minimize any lag in the start of the reaction. At each of five or more designated time points, 50 μl was removed and added to a prechilled tube on ice containing 0.5 ml of 12% trichloroacetic acid in order to terminate the reaction. The methylated RNAs collected by centrifugation were washed twice with 1.25 ml of ice-cold 6% trichloroacetic acid. After drying, the precipitate was extracted with 3 ml of scintillation fluid (Ultima Gold; Packard) and transferred to a counting vial. The remaining precipitate was extracted again with 75 μl of double-distilled water (DDW) warmed to 50°C to 60°C and then extracted once again with 25 μl of prewarmed DDW. All the extracts were pulled together, mixed well, and counted (Tri-Carb 2900TR; Packard, Shelton, CT, USA). A methylation assay at a single time point was carried out in accordance with the procedure described above, except for the reaction volume being 50 μl and the incubation time being 1 h. Experiments were repeated at least thrice. Methylation using chemically synthesized ribooligonucleotides was carried out as follows: 60 pmol of top strand (5′-AGGACGGA-3′) and bottom strand (5′-CCUAUCC-3′) was dissolved in 2 μl of self-folding buffer and annealed by sequential incubation at 50°C for 5 min and at 10°C for 90 min. Annealed duplex RNA was used for the methylation assay as described above, except for differences in the incubation temperature (18°C) and increased incubation time (1.5, 3, 6, 9, 12, and 15 h). To confirm the positive substrate activity obtained with a general methylation experiment, the levels of all the reaction components were doubled, namely, 120 pmol annealed ribonucleotides and ErmS enzyme, and 39.6 pmol SAM, and the methyl group-accepting activity was measured. By observing the concomitant increase in radioactivity from the substrate with increasing incubation time in both experiments, the substrate activity could be ensured. To confirm the specific methylation at A2058, two upper strand ribooligonucleotides containing A2058C and A2058U were used for detecting their methyl group-accepting activities.
